# Age-Related Differences in Affective Norms for Chinese Words (AANC)

**DOI:** 10.3389/fpsyg.2021.585666

**Published:** 2021-04-15

**Authors:** Pingping Liu, Qin Lu, Zhen Zhang, Jie Tang, Buxin Han

**Affiliations:** ^1^CAS(Chinese Academy of Sciences) Key Laboratory of Mental Health, Center on Aging Psychology, Institute of Psychology, Beijing, China; ^2^Department of Psychology, University of Chinese Academy of Sciences, Beijing, China; ^3^Department of Computing, The Hong Kong Polytechnic University, Hong Kong, China

**Keywords:** affective norms, ratings, age differences, Chinese, positivity effect

## Abstract

Information on age-related differences in affective meanings of words is widely used by researchers to study emotions, word recognition, attention, memory, and text-based sentiment analysis. To date, no Chinese affective norms for older adults are available although Chinese as a spoken language has the largest population in the world. This article presents the first large-scale age-related affective norms for 2,061 four-character Chinese words (AANC). Each word in this database has rating values in the four dimensions, namely, valence, arousal, dominance, and familiarity. We found that older adults tended to perceive positive words as more arousing and less controllable and evaluate negative words as less arousing and more controllable than younger adults did. This indicates that the positivity effect is reliable for older adults who show a processing bias toward positive vs. negative words. Our AANC database supplies valuable information for researchers to study how emotional characteristics of words influence the cognitive processes and how this influence evolves with age. This age-related difference study on affective norms not only provides a tool for cognitive science, gerontology, and psychology in experimental studies but also serves as a valuable resource for affective analysis in various natural language processing applications.

## Age-Related Differences in Affective Norms for Chinese Words (AANC)

Affective ratings of words are in high demand because they serve as valuable resources when studying emotions and cognition (Warriner et al., 2013; Kuperman et al., 2014; Yu et al., 2016; Ferrari et al., 2017; Stadthagen-Gonzalez et al., 2017; Kratzwald et al., 2018). With the aging global population, the role of age in modulating the processing of emotion information has become a focus of interest in different fields, especially in life span developmental psychology (Stine-Morrow et al., 2006; English and Carstensen, 2014; Notthoff and Carstensen, 2014; Reed et al., 2014; Steenhaut et al., 2018). Because of life experience and age-related biological changes, it is intuitive to speculate that differences in the perception of words by age should exist in terms of affective polarity, arousal, and control. However, to date, such age-normative information remains scarce, especially for Chinese. Little is known about age-related differences in the perception and meaning of affective words in Chinese that has the largest spoken language population in the world. Here, we attempted to close this gap by providing a new affective lexicon as a database with age-related differences in affective norms for Chinese words (AANC). AANC can be used as an age-adapted tool for future research on the processing of emotional words.

Most studies about affective norms have been based on Bradley and Lang's (1999) Affective Norms for English Words (ANEW) database. ANEW was developed within the dimensional theory of emotions (Wundt, 1912/1924; Osgood et al., 1957; Russell, 2003). The database contains ratings in three dimensions of ratings, namely, valence, arousal, and dominance. Valence refers to the degree of pleasantness elicited by a stimulus (ranging from unhappy to happy). Arousal reflects the subjective level of activation or intensity that a stimulus evokes (ranging from calm/quiet to excited/active). Dominance refers to the degree of control exerted by a stimulus (ranging from weak/submissive to strong/dominant). Based on Bradley and Lang's (1999) procedure, affective norms of words are available in a number of languages, including English (Stadthagen-Gonzalez and Davis, 2006; Eilola and Havelka, 2010; Warriner et al., 2013; Scott et al., 2019), French (Gilet et al., 2012; Monnier and Syssau, 2017), German (Grühn and Smith, 2008; Kanske and Kotz, 2011; Schmidtke et al., 2014), Spanish (Ferré et al., 2017; Stadthagen-Gonzalez et al., 2017; Sabater et al., 2020), Portuguese (Soares et al., 2012), Dutch (Moors et al., 2013), Polish (Imbir, 2015), Italian (Montefinese et al., 2014), and Chinese (Wang et al., 2008; Yu et al., 2016; Yao et al., 2017; Liu et al., 2018). Although most affective norms are rated by young adults, these works have clearly demonstrated that affective meanings of words vary with languages and cultures.

## Age-Related Differences in Emotional Functions

In addition to language differences, affective meanings of words seem to vary with age. Age-related differences in emotional context have been evidenced when words were rated by children and adults (Monnier and Syssau, 2017; Vesker et al., 2018, 2020; Morningstar et al., 2019; Sabater et al., 2020). The available evidence suggested that young children's ratings of valence were more extreme than those of adolescents and adults (Monnier and Syssau, 2017; Vesker et al., 2018). Furthermore, youngest children considered more words to be positive than adolescents (Sabater et al., 2020). These findings have confirmed that the affective evaluations of information vary with age. Such findings demonstrate the importance of providing age-adapted tools to researchers so they may explore, from a developmental point of view, how affective words are processed.

However, studies on how emotional stimuli are processed by older adults remain scarce. Can valence, arousal, and dominance ratings of younger adults be generalized to older adults? Studies on aging and emotions have indicated that older adults and younger adults indeed differ in several aspects of emotional functions (Mather and Carstensen, 2005; English and Carstensen, 2014; Notthoff and Carstensen, 2014; Wirth et al., 2017; Steenhaut et al., 2018). This has been considered within the context of socioemotional selectivity theory (Carstensen, 2006). As older adults view their lifetime as limited, they prioritize present-focused goals related to emotional meaning and satisfaction. Emotional experience appears to grow more positive with age, and this refers to the well-documented positivity effect. First, older adults attend more to positive information and less to negative information compared to younger adults (Mroczek and Kolarz, 1998; Kunzmann et al., 2000; Carstensen, 2006; English and Carstensen, 2014). Second, older adults tend to report having better developed emotion regulation abilities than younger adults do, and they also appear to dissipate negative affect more effectively (Grühn and Scheibe, 2008; Hess et al., 2013). Third, older adults tend to show reduced autonomic reactions to emotional stimuli compared to younger adults (Keil and Freund, 2009; Uchino et al., 2010; Streubel and Kunzmann, 2011; Ferrari et al., 2017; Steenhaut et al., 2018). Overall, a meta-analysis by Reed et al. (2014) has confirmed that older adults show a significant information processing bias toward positive vs. negative information, whereas younger adults show the opposite pattern. Thus, these age-related differences in emotional experience, control, and reactivity suggest that emotional ratings of younger adults could not be generalized to older adults.

Evidence regarding age-related differences in subjective evaluations of emotional words remains scarce, although there are several age-related studies available mainly for pictorial materials. Some previous studies have obtained emotional ratings of standardized pictures from the International Affective Picture System (IAPS; Lang et al., 1998) between older and younger adults with inconsistent results. Some studies reported that older adults showed lower subjective ratings of their feelings than younger adults (Keil and Freund, 2009; Streubel and Kunzmann, 2011). However, other studies demonstrated that older adults showed higher subjective ratings (Gavazzeni et al., 2008; Grühn and Scheibe, 2008; Steenhaut et al., 2018) or that ratings between older and younger adults were similar (Wieser et al., 2006; Ferrari et al., 2017). Possible mechanisms under these age-related inconsistencies have not been well-established (Steenhaut et al., 2018). Furthermore, some studies have revealed age-related differences in the neural processing of emotional pictures and words (Kensinger and Schacter, 2006; Leclerc and Kensinger, 2011). Understanding other types of age-related emotional stimuli, especially for words, can bring some clarity to age-related differences in emotional reactivity.

Although changes of affective responses to words between older and younger adults have been reported in German (Grühn and Smith, 2008; Keil and Freund, 2009), French (Gilet et al., 2012), English (Ready et al., 2017), Finnish (Söderholm et al., 2013), and Italian (Fairfield et al., 2017), they show mixed results on age-related differences, especially for negative words. Grühn and Smith (2008) found that, compared with younger adults, older adults perceived negative words as less arousing and more controllable and evaluated positive words as more positive, more arousing, and less controllable. It could be considered as the positivity effect that indicates an age-related increase in the preference for positive over negative words in rating tasks (Söderholm et al., 2013). However, other studies demonstrated that older adults tended to rate negative words as more arousing than younger adults did (Gilet et al., 2012; Ready et al., 2017). Thus, age-related differences in affective meanings of words, especially for negative words, appear to vary in different languages such as German and English. Furthermore, there are some differences between Western and Eastern cultures, such as personality, culture, and social relationships (Markus and Kitayama, 1991; Fung, 2013). Therefore, age-related differences in affective ratings demonstrated in Western cultures may not be generalizable to Eastern cultures such as Chinese.

## Limitations of Current Chinese Affective Norms

To our knowledge, no Chinese affective norms for older adults have been available so far. Although a large proportion of world population consists of older adults in China, little is known about age-related differences in subjective evaluations of emotional words in Chinese. Additionally, most existing Chinese affective norms provide valence, arousal, and/or dominance ratings for two- or three-character words obtained from younger adults (Wang et al., 2008; Lei and Zhang, 2013; Yu et al., 2016; Yao et al., 2017). However, few Chinese affective lexicons are based on four-character words, which convey more complex and abundant meanings than two- and three-character words. According to the Chinese Lexicon (2003), 63.9% of words are two-character words, 17.5% are three-character words, and 14.2% are four-character words. Some four-character words are idioms that have fixed expressions and cannot be derived directly from the constitute words (e.g., 寿比南山/shou4bi3nan2shan1/longevity; Li et al., 2016). Recently, Liu et al. (2018) reported an annotated dataset for four-character words on valence and arousal rated by younger adults. However, this database did not provide ratings on the dominance dimension, which is regarded as an important variable in emotion studies (Osgood et al., 1957; Fontaine et al., 2007; Warriner et al., 2013). In sum, there is no database that has affective ratings for older adults, and the affective lexicon for four-character words remains scarce in Chinese.

## The Present Study

In order to address the research gap on age-related differences in evaluations of affective words in Chinese, we provide the first large-scale affective norms with well-designed procedures in the lab for 2,061 four-character words rated on four dimensions (valence, arousal, dominance, and familiarity) from both older and younger adults. Our norms contain ratings of familiarity that reflect how well-known a given stimulus is. Familiarity with a given stimulus would presumably make processing easier, so that researchers should consider familiarity when designing experiments addressing the processing of affective language (Hinojosa et al., 2016). Although familiarity is related to word frequency, it has been found to be a better predictor of performance than frequency (Gordon, 1985; Kuperman and Dyke, 2013). Therefore, familiarity ratings can be used as a complement to word frequencies for both older and younger adults. The common use and definitions of “older adult” vary in the literature, and there are different chronological cut points for older adulthood in previous studies (55+, 60+, or 65+; Lawrence and Singleton, 2017; Sinclair and Grieve, 2017). In order to effectively ground the current study with relevant literature, we defined “older adult” as being aged 55 years or older (Sinclair and Grieve, 2017; DeCaro and Thomas, 2019). We defined “younger adults” as being aged 16 years or older with an upper age limit of 40 (Murphy and Isaacowitz, 2008). We expected that there would be age-related differences in subjective evaluations of emotional words, especially for negative words (Grühn and Smith, 2008; Gilet et al., 2012; Ready et al., 2017). According to the positivity effect that older adults favored processing of pleasant stimuli (Carstensen, 2006; English and Carstensen, 2014; Reed et al., 2014), we anticipated that, compared with younger adults, older adults should show greater attention to positive words vs. negative words. Older adults might have increased emotional reactions to positive words and have reduced emotional reactions to negative words.

We aimed to provide a set of age-related differences in affective norms. This collection is the largest published database reporting older adults' assessment of the emotional properties of words in Chinese so far. We consider that this collection can contribute to the academic community in at least three aspects. First, the collection of four-character words can serve as a supplemental resource to the currently available two-character affective lexicon for Chinese sentiment computing and emotional research. Second, the database can provide a tool for cognitive science and gerontology in experimental studies. Finally, this database can serve as raw data to enable researchers to study how emotion influences cognitive processing and how this influence evolves with age.

## Method

### Participants

One hundred twenty-five older adults (*M* = 70.52 years, *SD* = 5.90 years, 56–85 years of age, 50.4% female) and 160 younger adults (*M* = 21.58 years, *SD* = 3.40 years, 16–40 years of age, 50% female) from the local community and university campus were recruited through advertisements in Beijing for this study. All participants had at least 12 years of formal education and were native Chinese speakers with normal or corrected-to-normal vision. They received an honorarium of 50 *RMB* per hour for their participation. The study was approved by the institutional review board of the Institute of Psychology, Chinese Academy of Sciences. Forty-five percent of the younger cohort was the same as those described in Liu et al. (2018). This study recruited additional younger adults and the new older cohort in order to collect sufficient data to study age differences.

In order to screen for possible mild cognitive impairment, all older participants were given the Mini Mental State Examination (MMSE) as a preliminary screening measure, and the minimum score of 26/30 was required (Folstein et al., 1975). This test was used as an indication that the participants had intact cognitive abilities to perform the word rating task. Then, all participants' demographic details and their self-rated health information were collected. Seven older adults and one younger adult were removed because of their low education or lower scores in neuropsychological tests. Three older adults were removed because they could not use a computer to complete the rating task. One older adult and nine younger adults were removed due to a high number of outlier scores because their ratings seemed to be given at random. Only three older participants were under 60 years of age. Reanalyses excluding the three youngest of our older participants did not change any statistical conclusions in the study. Thus, the final sample consists of 114 older adults (*M* = 70.05 years, *SD* = 6.01 years, 97.37% of the older participants were over 60 years old, range = 56–84 years; 54% female; *M*_*MMSE*_ = 29.24 ± 0.91) and 150 younger (*M* = 21.59 years, *SD* = 3.41 years, 90.67% of the younger participants were aged between 18 and 29 years, range = 16–38 years; 50% female), and they were free from neurological and psychiatric disorders.

## Materials

Three graduate students who have good linguistics knowledge were assigned to select a set of four-character words that were considered frequently used and some might trigger their subjective feelings (positive, neutral, or negative). A set of 2,290 four-character words were selected from the Chinese Lexicon (2003). Here, 229 words were removed due to typographical errors or unfamiliarity. The mean word frequency of the final 2,061 words was 135 (*SD* = 259, range = 2–5,384, median = 69) occurrences per million, and the mean word complexity of the set was 30.63 (*SD* = 7.42, range = 8–72, median = 30). These indicated that the 2,061 words could be considered frequently used. The average frequencies of the first, second, third, and fourth characters were 1,076, 1,241, 1,277, and 1,160 occurrences per million, respectively. The average complexity of the first, second, third, and fourth characters were 7.76, 7.50, 7.58, and 7.78, respectively. For parts-of-speech tag distribution, our set contains 33.77% idioms (696), 17.86% adjectives (368), 17.61% nouns (363), 15.04% verbs (310), and 15.72% of all words (324) that have two or more parts-of-speech tags (e.g., idioms, adjectives, and adverbs).

## Procedure

For each word, each dimension was rated by a minimum of 48 participants (24 older adults, 24 younger adults). The four dimensions of these 2,061 words were designed into two versions of a questionnaire (one for valence and arousal, another for dominance and familiarity). After the rating task, an option to mark a word as Unknown (one for unknown, two for known) in an Excel file was given to each participant. The first version was completed by 99 older participants (*M* = 70.52 years, *SD* = 6.08 years; 53% female) and 102 younger participants (*M* = 21.85 years, *SD* = 3.63 years; 50% female) who rated the words in terms of valence and arousal. These data were obtained between May and August in 2015. The second version of the questionnaire was completed by 46 older participants (*M* = 70.22 years, *SD* = 5.61; 52% female) and 78 younger participants (*M* = 21.09 years, *SD* = 2.59; 50% female) who rated the words in terms of dominance and familiarity. These data were collected between 2017 and 2018. Thirty-one older participants and 30 younger participants completed both the first and second versions of the questionnaire.

The 2,061 words were distributed across six blocks containing 343–344 words in each block for older participants. Five blocks containing 412–413 words were given to younger participants, given that older adults respond slower than younger adults (Stine-Morrow et al., 2006; Paterson et al., 2013; Rayner et al., 2014; Shafto and Tyler, 2014; Liu et al., 2015). To avoid primacy or recency effects, the order in which words appeared in the block was randomized across participants. Participants can choose to rate up to five blocks at their convenience, and they were asked to leave at least a 6-h interval between two blocks. The order of these blocks was counterbalanced across participants.

**Figure 1:**
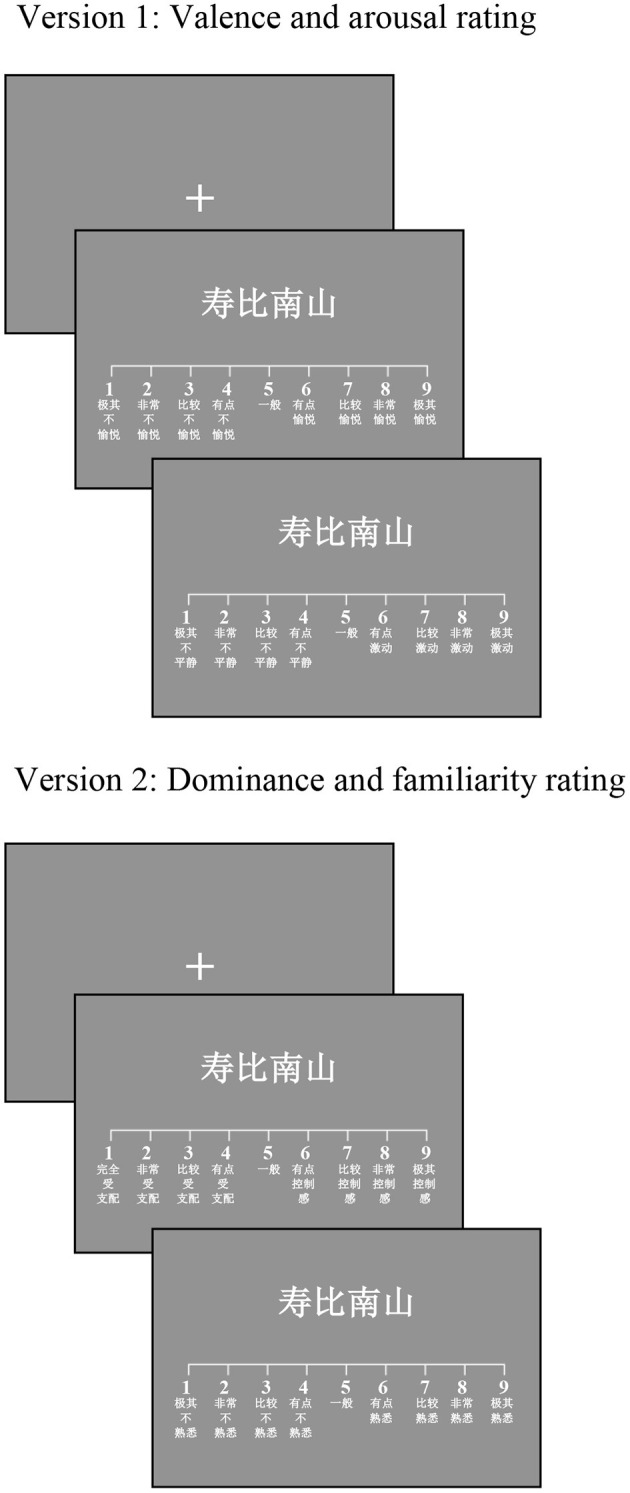
An example of the paradigm used in the study to explore the ratings of relevant dimensions for the 4-character word 寿比南山(/shou4bi3nan2shan1/, longevity). Each trial began with a fixation cross (+) displayed for 600 ms. Then, the given word and the respective nine-point scale were presented until participants responded to make their rating using the computer mouse. In data collection of version one, participants rated first for valence and then for arousal. In data collection of version two, participants first rated for dominance and then for familiarity. Response scales ranged from extremely unpleasant/calming/controlled/unfamiliar (1) to extremely pleasant/exciting/control/familiar (9) for the four dimensions.

A computer-based questionnaire was used. Participants gave the ratings in the lab at the Institute of Psychology of Chinese Academy of Sciences, Beijing, in small age-homogeneous groups of 2–6 persons in the presence of two researchers. After completing informed consent, some demographic questions (i.e., age, gender, education, and self-reported health) and the MMSE test were collected. Each participant sat in front of a desktop computer and received an instruction sheet for the relevant dimensions (i.e., valence/arousal or dominance/familiarity) before starting the rating procedure. All dimensions were rated on nine-point scales (Figure 1). At the beginning of the rating procedure, participants were given instructions with examples and the opportunity to practice 15 trials using the scale to ascertain that they understood the task. The instructions were either adapted on the basis of the original instructions taken from previous published studies (Bradley and Lang, 1999; Stadthagen-Gonzalez and Davis, 2006; Eilola and Havelka, 2010; Warriner et al., 2013; Stadthagen-Gonzalez et al., 2017) or from previous Chinese normative studies (Wang et al., 2008; Yao et al., 2017; Liu et al., 2018). The exact wording in Chinese and an English translation are provided available online (see the Supplemental Material).

The paradigm was automated using E-prime (Psychology Software Tools, Inc., Sharpsburg, PA), and stimuli were presented on a computer display. As shown in Figure 1, each trial began with a fixation cross (+) displayed in the center of the screen for 600 ms. Each word was displayed, one at a time along with the respective nine-point scale, until participants responded by clicking on the appropriate rating using the computer mouse. Word stimuli were presented on a 17-inch LCD monitor (resolution: 1,024 × 768 pixels, refresh rate: 85 Hz) in white on a light gray background. The contrast was low to minimize eye fatigue. Each word was displayed on a single line in Courier New 34-point font, and the size of each Chinese character was 84 × 84 pixels. Participants were allowed to stop rating and to resume after a short break at their own pace. The rating task lasted about an hour for older participants and 45 minutes for younger participants.

## Results and Discussion

### Data Trimming

Altogether, 496,712 data points of ratings were collected across all four dimensions. We conducted the following outlier analysis. First, we removed 214 words that were marked as Unknown by more than 10% of the participants according to previous studies (Soares et al., 2012; Moors et al., 2013; Warriner et al., 2013; Montefinese et al., 2014; Stadthagen-Gonzalez et al., 2017). We also discarded all ratings with which participants indicated that the word was unknown to them (3.3% of all). Second, we discarded ratings of participants who gave the same rating for more than 85% of the words for each dimension (0.61%). Third, we excluded the ratings for 15 words due to typographical errors (0.069%). Fourth, means and standard deviations (*SDs*) were calculated for each word of older and younger participants, respectively. We removed the data points for those participants whose scores were 2.5 *SDs* away from their group's average ratings for each word (3.2% of all). This resulted in the final set of 2,061 words consisting of 130,960 observations for valence and arousal separately (91% of the original data pool) and 100,775 observations for dominance and familiarity separately (96% of the original data pool).

### Description of AANC Database

In the final data set, 99.98% of the 2,061 words were rated by at least 20 older adults and 20 younger adults for each dimension. For each word, we calculated the mean and *SD* for each age group and compiled the affective ratings and familiarity into a database. The database contains 2,061 entries for the corresponding Chinese words based on Romanized Pinyin order, together with their English translations (based on Google Translation, Baidu Translation, and five Chinese-English bilinguals), mean rating values, and sample sizes (number of participants). Mean rating values (Mean) and *SD* of the four dimensions for each word are given for the global samples (All), the older adults, and the younger adults, respectively (see the Supplemental Material available online). The AANC database also contains information about word frequency, word complexity, character frequency, and character complexity, which were taken from the Chinese Lexicon (2003).

### Descriptive Statistics

Table 1 shows descriptive statistics and group differences for valence, arousal, dominance, and familiarity ratings for each age group. Younger adults rated words significantly higher than older adults for dominance [*t*_(2,060)_ = 19.26, *p* < 0.001, *Cohen's d* = 0.33] and familiarity [*t*_(2,060)_ = 35.01, *p* < 0.001, *Cohen's d* = 0.76], while older adults rated words slightly higher than younger adults for arousal [*t*_(2,060)_ = 6.68, *p* < 0.001, *Cohen's d* = 0.12]. These results showed that younger adults tended to rate words more in control and more familiar than did older adults. No age differences were found in mean ratings for valence (*p* = 0.384). The correlations between older and younger adults' ratings for the 2,061 words were extremely high for valence (*r* = 0.95, *p* < 0.001) but lower for the arousal (*r* = 0.73, *p* < 0.001), dominance (*r* = 0.62, *p* < 0.001), or familiarity (*r* = 0.53, *p* < 0.001) dimensions. It revealed that older and younger adults agreed on whether a word was positive or negative. The ratings of arousal, dominance, and familiarity might involve more individual and heterogeneous responses than valence.

**Table 1:** Descriptive statistics and group differences for valence, arousal, dominance, and familiarity ratings by age.

	**Older**					**Younger**					
**Dimension**	**Mean**	***AvgSD***	**Min**	**Max**	**Range**	**Mean**	***AvgSD***	**Min**	**Max**	**Range**	***p***
Valence	4.89	1.10	1.35	8.07	6.72	4.88	1.11	1.79	7.95	6.16	0.384
Arousal	5.63	1.37	4.29	8.00	3.71	5.54	1.50	3.00	7.83	4.83	<0.001
Dominance	4.72	1.74	2.17	6.83	4.66	5.07	1.69	2.05	7.68	5.63	<0.001
Familiarity	6.84	1.22	4.78	8.04	3.26	7.20	1.37	4.44	8.42	3.98	<0.001

*Notes. Reported are the group means of ratings, the average standard deviations (AvgSD), the minimum (Min), the maximum (Max), the range of the average rating means, and, in the last column, the p-value of a two-tailed paired t test comparing the group means*.

**Figure 2:**
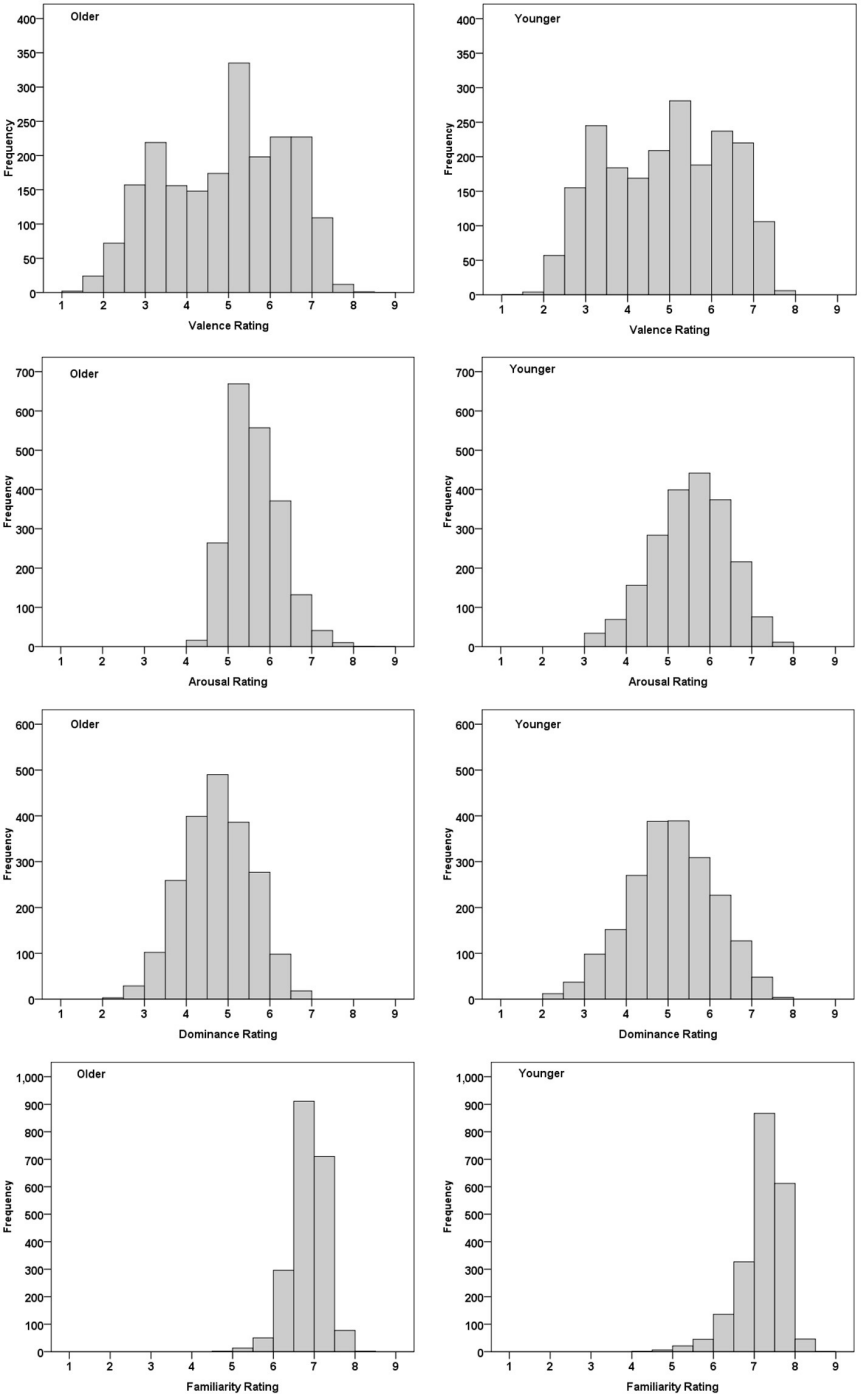
Distribution of valence, arousal, dominance, and familiarity ratings for the older and younger adults. Each bar represents the number of words rated within one interval of the scale.

Figure 2 shows the distributions of each dimension ratings for older and younger adults. Consistent with prior reports (Fairfield et al., 2017; Kurdi et al., 2017), the eight distributions deviated significantly from a normal distribution (Kolmogorov–Smirnov test: *Ds* > 0.020, *ps* < 0.05). The distributions of valence, dominance, and familiarity ratings were negatively skewed for both older (*G*_1_*s* = −0.20, −0.069, and −0.69, respectively) and younger adults (*G*_1_*s* = −0.092, −0.14, and −1.29, respectively). Arousal was positively skewed for older (*G*_1_ = 0.60) but negatively skewed for younger adults (*G*_1_ = −0.24). Older adults' arousal responses were distributed in a smaller range (4.5–7.0) than that of the younger adults (3.5–7.5). For older and younger adults, percentages of words rated above the middle of the arousal rating scale (the score of 5.0) were 84% and 72% (significant age effects: χ^2^ = 82.63, *p* < 0.001). Here, 36% and 52% of all words were rated above the middle of the dominance rating scale for older and younger adults, respectively (significant age effects: χ^2^ = 104, *p* < 0.001). These results indicated that younger adults tended to consider the words as more controllable and less exciting compared to older adults.

**Figure 3:**
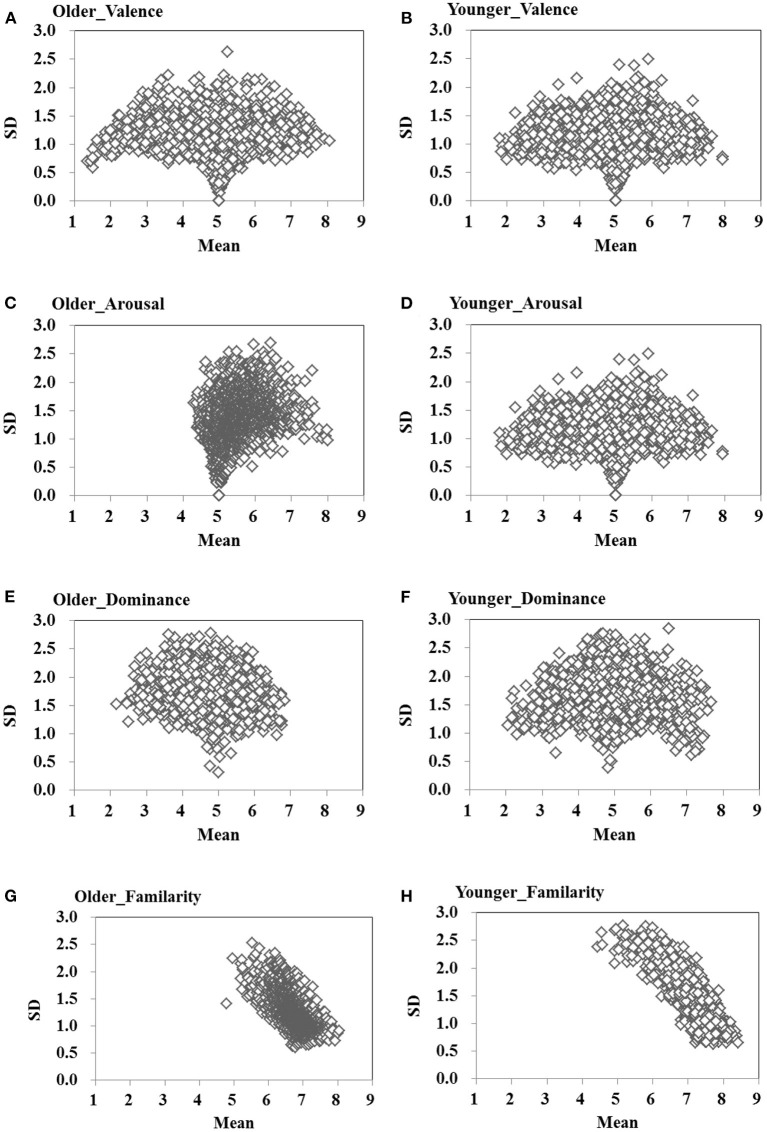
Average standard deviations (variance among responders) across the valence **(A,B)**, arousal **(C,D)**, dominance **(E,F)**, and familiarity **(G,H)** ranges per word for older and younger adults.

Figure 3 shows plots of the means and *SDs* of the ratings for all dependent variables for older and younger adults. Valence ratings were relatively stable across participants (*SD*_*Avg*_ <1.20), while arousal, dominance, and familiarity were much more divergent (*SD*_*Avg*_ > 1.22; Table 1). This was also indicated by the difference between the average *SDs* of the dimensions from the global sample: 1.13 for valence, 1.48 for arousal, 1.77 for dominance, and 1.33 for familiarity.

For valence, similar to the patterns reported by Moors et al. (2013), the scatterplot (Figures 3A,B) shows that there were two types of words in the midrange: (a) words with low *SDs* upon which most participants agreed that they were neutral, such as the word for 南回归线(/nan2hui2gui1xian4/, Tropic of Capricorn), and (b) words with high *SDs* that evoked both rating values of opposite polarity by different participants. For example, the word for 慷慨就义(*/*kang1kai3jiu4yi4/, go to one's death like a hero) was rated as negative by 37% and positive by 41% of all participants, respectively. We also found similar age-related patterns in average *SDs* for valence, *t*_(2,060)_ = 0.64, *p* = 0.53, *Cohen's d* = 0.01. The scatterplot was symmetrical at the median (Figures 3A,B), and this indicates that relative positive or negative words are associated with smaller variability in the ratings across participants compared to valence-neutral words (Moors et al., 2013; Warriner et al., 2013; Stadthagen-Gonzalez et al., 2017).

The scatterplots for arousal (Figure 3D) and dominance (Figures 3E,F) were somewhat similar to that for valence, but less pronounced. Older adults' scatterplot for arousal showed that the *SD* increased with increasing means (Figure 3C). For both older and younger adults, the scatterplot of familiarity (Figures 3G,H) showed that *SD* decreased with increasing means. It showed that there was more consensus on familiar words than unfamiliar words. A series of paired *t-test* on *SD* indicated that the arousal [*t*_(2,060)_ = 11.11, *p* < 0.001, *Cohen's d* = 0.34] and familiarity [*t*_(2,060)_ = 15.47, *p* < 0.001, *Cohen's d* = 0.40] analysis showed more variability for younger adults than for older adults, whereas dominance analysis showed more variability for older adults than for younger adults [*t*_(2,060)_ = −5.48, *p* < 0.001, *Cohen's d* = −0.14]. Overall, these results indicate that the perceived valence of words tends to generalize well (Eilola and Havelka, 2010; Soares et al., 2012; Moors et al., 2013; Warriner et al., 2013), whereas the ratings of arousal, dominance, and familiarity show greater variability for both groups.

### Reliability and Validity of the Norms

We explored the interrater reliability of the four ratings with a split-half procedure. We randomly split the participants who rated each word into two equal groups and calculated their mean ratings for each word. After computing the correlations between these two groups of participants, we repeated this task 10 times to get a set of 10 correlations. These steps were also repeated for each age group. For all participants, the mean correlations between the two equal groups were very high for valence (*r* = 0.988, *p* < 0.001), arousal (*r* = 0.916, *p* < 0.001), dominance (*r* = 0.906, *p* < 0.001), and familiarity (*r* = 0.783, *p* < 0.001). The split-half reliabilities for both older and younger groups are based on smaller halves than those for all participants, and this may explain why the former are sometimes smaller than the latter (see Table 2 for details). Regarding these affective variables, valence had a higher interrater reliability than arousal and dominance ratings (Moors et al., 2013; Ferré et al., 2017; Monnier and Syssau, 2017; Yao et al., 2017). Our results show that the ratings are highly reliable and can be used across the Chinese-speaking population.

**Table 2:** Means (M) and range for the interrater split-half reliabilities for each dimension.

**Dimension**	**All participants**		**Older**		**Younger**	
	**M**	**Range**	**M**	**Range**	**M**	**Range**
Valence	0.988	0.987–0.989	0.977	0.977–0.978	0.975	0.974–0.977
Arousal	0.916	0.911–0.921	0.800	0.793–0.812	0.887	0.877–0.895
Dominance	0.906	0.902–0.909	0.804	0.796–0.818	0.879	0.874–0.884
Familiarity	0.783	0.770–0.792	0.624	0.609–0.649	0.703	0.689–0.728

Apart from reliability, it was necessary to evaluate the validity of the norms. A common approach is to compare these values, when possible, with those obtained from other resources. To our knowledge, it should be noted that there have been no normative data for older adults in Chinese until now. All ratings of valence, arousal, and dominance for older adults in the present database were novel, and we could not precisely compare the ratings by older adults to other resources. However, some words in our database had already been rated in previous studies. This allowed us to assess the validity of our ratings by comparing them with those of the normative studies with overlapping words for both older and younger adults. For the affective ratings of the overlapping words, there was high correlation between our values and those of CVAW words (Yu et al., 2016) for both valence [*r*_*older*_ (10) = 0.94, *p* < 0.001, *r*_*younger*_ (10) = 0.92, *p* < 0.001] and arousal [*r*_*older*_ (10) = 0.54, *p* < 0.001, *r*_*younger*_ (10) = 0.63, *p* = 0.015]. Correlations were also high with the ratings of Li et al. (2016) for familiarity [*r*_*older*_ (48) = 0.32, *p* = 0.025, *r*_*younger*_ (48) = 0.54, *p* < 0.001]. Similarly, correlations were high with the ratings of Liu et al. (2018) for both valence [*r*_*older*_ (1,993) = 0.94, *p* < 0.001, *r*_*younger*_ (1,993) = 0.99, *p* < 0.001] and arousal [*r*_*older*_ (1,993) = 0.71, *p* < 0.001, *r*_*younger*_ (1,993) = 0.98, *p* < 0.001].

We also translated the words of our list into English and found 711 one-word translation equivalence in common with those in the list of Scott et al. (2019). Pearson correlations were high in valence [*r*_*older*_ (710) = 0.77, *p* < 0.001, *r*_*younger*_ (710) = 0.80, *p* < 0.001]. However, the correlation between Chinese and English in arousal [*r*_*older*_ (710) = 0.34, *p* < 0.001, *r*_*younger*_ (710) = 0.28, *p* < 0.001], dominance [*r*_*older*_ (710) = 0.21, *p* < 0.001, *r*_*younger*_ (710) = 0.51, *p* < 0.001], and familiarity [*r*_*older*_ (710) = 0.15, *p* < 0.001, *r*_*younger*_ (710) = 0.09, *p* = 0.014] were lower. The results of the correlation analyses showed a high rating consistency in the context of Chinese (Li et al., 2016; Yu et al., 2016; Liu et al., 2018) but not for English (Scott et al., 2019). These results help to reinforce the argument of affective differences between words in different languages. This suggests that affective ratings must be done on language basis and affective resources could not be directly used through simple translation.

## Correlations Between Dimensions

### Global Sample Analysis

**Figure 4:**
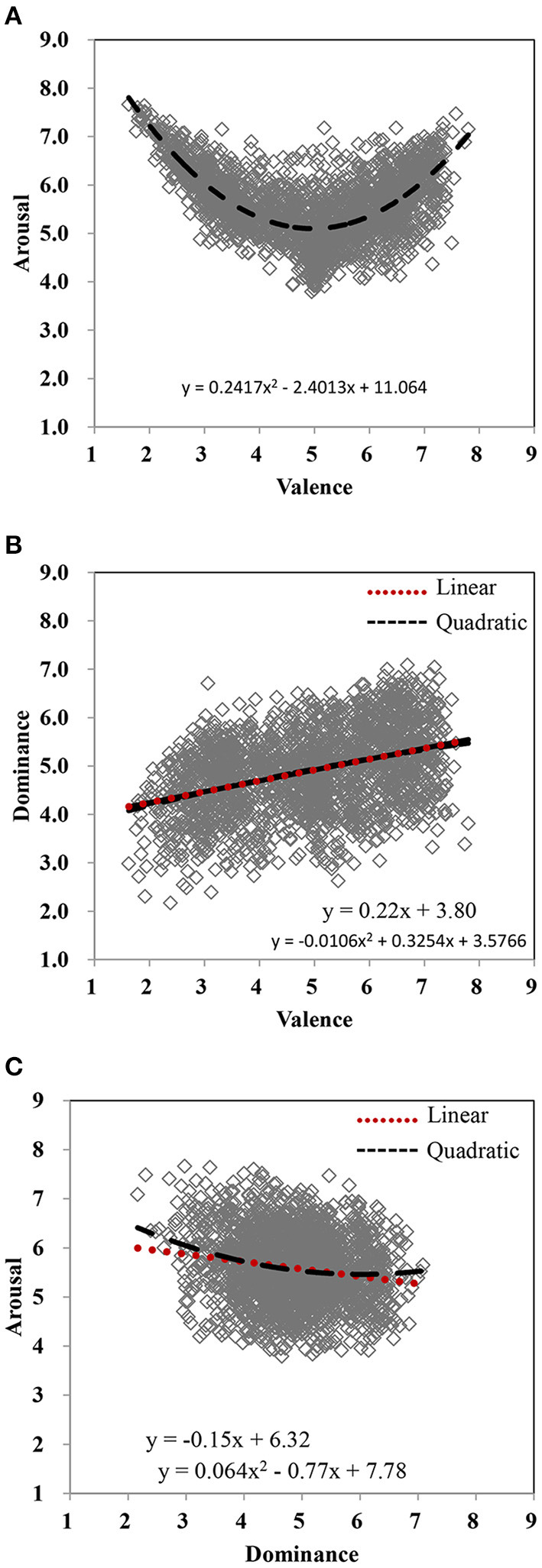
Scatterplots of dimensions for all 2,061 words [**(A)**, valence vs. arousal; **(B)**, valence vs. dominance; **(C)**, dominance vs. arousal]. The linear and quadratic associations between dimensions are represented by red dotted and black dashed lines, respectively.

Pearson's correlations, linear and quadratic fits, and the test for the increase of *R*^2^ were calculated between dimensions (Table 3). First, valence and arousal showed the typical U-shaped relationship (Figure 4A), which were highly consistent with prior studies (Bradley and Lang, 1999; Eilola and Havelka, 2010; Soares et al., 2012; Warriner et al., 2013; Schmidtke et al., 2014; Stadthagen-Gonzalez et al., 2017; Yao et al., 2017; Liu et al., 2018). The quadratic relationship between valence and arousal was significant (*R*^2^ = 0.47, *p* < 0.001) and outperformed (Δ*R*^2^ = 0.44, *p* < 0.001) the linear relationship (*R*^2^ = 0.033, *p* < 0.001). Compared to valence-neutral words (e.g., 正三角形/zheng4san1jiao3xing2/, equilateral triangle), very positive words (e.g., 民富国强/min2fu4guo2qiang2/, the people are rich and the country is strong) or very negative ones (e.g., 丧子之痛/sang4zi3zhi1tong4/, bereavement of the son's pain) were more arousing. This was corroborated by the positive correlation between valence and arousal for positive words (mean valence rating >6; *r* = 0.40, *p* < 0.001) and the negative correlation between them for negative words (mean valence rating <4; *r* = −0.78, *p* < 0.001).

**Table 3:** Pearson correlations (r), linear and quadratic fits between dimensions, and the test for the increase of the R^2^ (Δ*R*^2^) for all, older, and younger adults.

		**Linear**			**Quadratic**					
	***r***	***R^**2**^***	***F***	***b***	***R^**2**^***	***F***	***b_**1**_***	***b_**2**_***	***ΔR^**2**^***	***ΔF***
**All**										
Val vs. Aro	−0.18^***^	0.033	69.24^***^	−0.089	0.47	908.8^***^	−2.40	0.24	0.44	1,690.80^***^
Dom vs. Aro	−0.18^***^	0.032	67.12^***^	−0.15	0.038	40.98^***^	−0.77	0.064	0.0067	14.37^***^
Val vs. Dom	0.39^***^	0.15	361.12^***^	0.22	0.15	181.32^***^	0.33	−0.011	0.00077	1.44
Fam vs. Val	0.23^***^	0.051	111.68^***^	0.77	0.089	101.05^***^	−12.81	1.00	0.038	85.83^***^
Fam vs. Aro	0.054^*^	0.0029	5.96^*^	0.09	0.013	13.03^***^	3.45	−0.25	0.0096	20.09^***^
Fam vs. Dom	0.31^**^	0.098	224.41^***^	0.62	0.13	157.16^***^	−6.87	0.55	0.034	81.39^***^
**Val vs. Aro**										
Older	−0.048^*^	0.0023	4.80^*^	−0.02	0.53	1,159^***^	−2.01	0.21	0.53	2,305.77^***^
Younger	−0.25^***^	0.062	136.02^***^	−0.16	0.38	624.34^***^	−2.73	0.27	0.32	1,043.07^***^
**Dom vs. Aro**										
Older	−0.10^***^	0.010	21.03^***^	−0.075	0.014	14.56^***^	−0.50	0.046	0.0037	7.95^**^
Younger	−0.25^***^	0.064	141.34^***^	−0.22	0.073	81.20^***^	−0.84	0.062	0.0089	19.73^***^
**Val vs. Dom**										
Older	0.11^***^	0.012	24.07^***^	0.06	0.013	13.27^***^	0.18	−0.013	0.0013	2.43
Younger	0.55^***^	0.301	886.76^***^	0.40	0.301	443.85^***^	0.49	−0.010	0	0.956
**Fam vs. Val**										
Older	0.30^***^	0.089	201.99^***^	1.06	0.12	142.88^***^	−11.18	0.91	0.033	76.48^***^
Younger	0.12^***^	0.015	31.39^***^	0.32	0.032	33.89^***^	−4.89	0.38	0.017	35.86^***^
**Fam vs. Aro**										
Older	0.075^**^	0.0056	11.61^***^	0.11	0.0058	6.01^**^	0.51	−0.030	0.00015	0.40
Younger	0.061^**^	0.004	7.81^**^	0.10	0.015	15.50^***^	2.76	−0.19	0.011	23.13^***^
**Fam vs. Dom**										
Older	0.26^***^	0.065	142.64^***^	0.50	0.071	78.47^***^	−2.43	0.22	0.0057	13.45^***^
Younger	0.23^**^	0.053	114.24^***^	0.44	0.088	99.31^***^	−5.03	0.40	0.035	79.90^***^

*Notes. Val, valence; Aro, arousal; Dom, dominance; Fam, familiarity*.

*** *p < 0.001,*

** *p < 0.01,*

* *p < 0.05*.

Second, as shown in Table 3 and Figure 4, dominance was positively associated with valence (*r* = 0.39), yet dominance was negatively associated with arousal (*r* = −0.18). The relationship between dominance and valence tended to be linear (Table 3, Figure 4B), but the linear and quadratic associations did not differ significantly (Δ*R*^2^ = 0.00077, *p* = 0.230). We also performed a linear and quadratic model with mean arousal and its square as independent variables and mean dominance as a dependent variable (Table 3, Figure 4C). The quadratic term best explained the relationship between dominance and arousal (Δ*R*^2^ = 0.0067, *p* < 0.001), although the effect was weak. Generally, words that made people feel happier also made them feel more in control (e.g., 胸怀坦荡/xiong1huai2tan3dang4/, magnanimous mind), and negative words made people feel less in control. Words that made people feel more in control were less arousing (e.g., 实心实意/shi2xin1shi2yi4/, honest and sincere), but words rated less dominant seemed to be more arousing (e.g., 天塌地陷/tian1ta1di4xian4/, earth crumbles).

**Figure 5:**
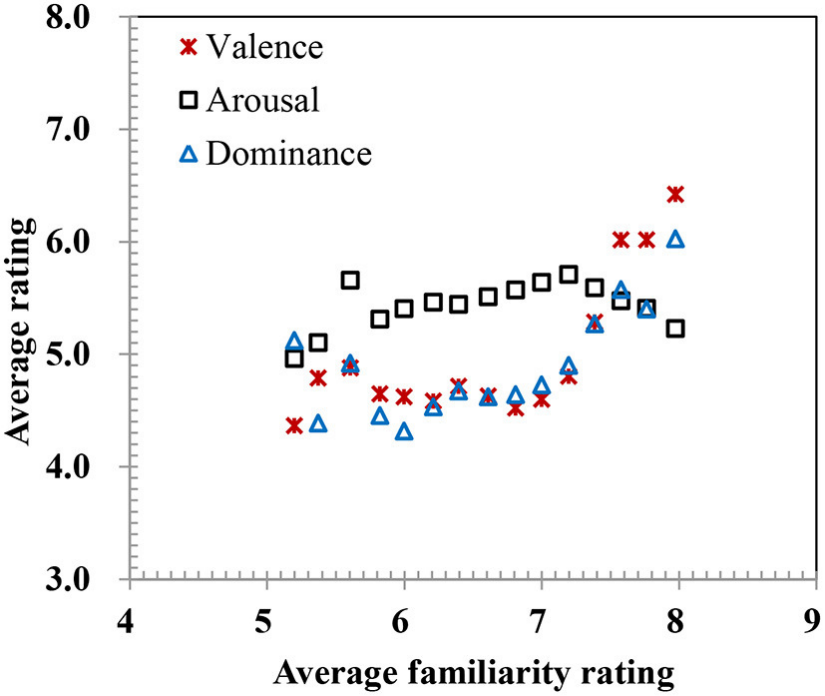
Relationships between affective dimensions and familiarity for overall average rating.

Third, familiarity had positive correlations with valence (*r* = 0.23), arousal (*r* = 0.054), and dominance (*r* = 0.31), although the relationships were non-linear (Table 3, Figure 5). Generally, words rated as more familiar were likely to be regarded as more positive and dominant. Finally, all these results of global analyses should be taken with caution because they may be mediated by age, which will be considered in detail in the following.

### Age Differences in Associations Between Dimensions

To examine the relationships between different dimensions and to test how age influences these relationships, we assessed associations between dimensions for older and younger adults using the Fisher *r-to-z* transformation (http://vassarstats.net/rdiff.html). There were significant age-related differences between the correlation coefficients for valence and arousal (*Z* = 6.67, *p* < 0.001), dominance and arousal (*Z* = −5.09, *p* < 0.001), valence and dominance (*Z* = −16.16, *p* < 0.001), as well as familiarity and valence (*Z* = 5.90, *p* < 0.001). Such difference was not obvious between the correlation coefficients for familiarity and arousal (*Z* = −1.48, *p* = 0.14) as well as familiarity and dominance (*Z* = 5.90, *p* = 0.29).

**Figure 6:**
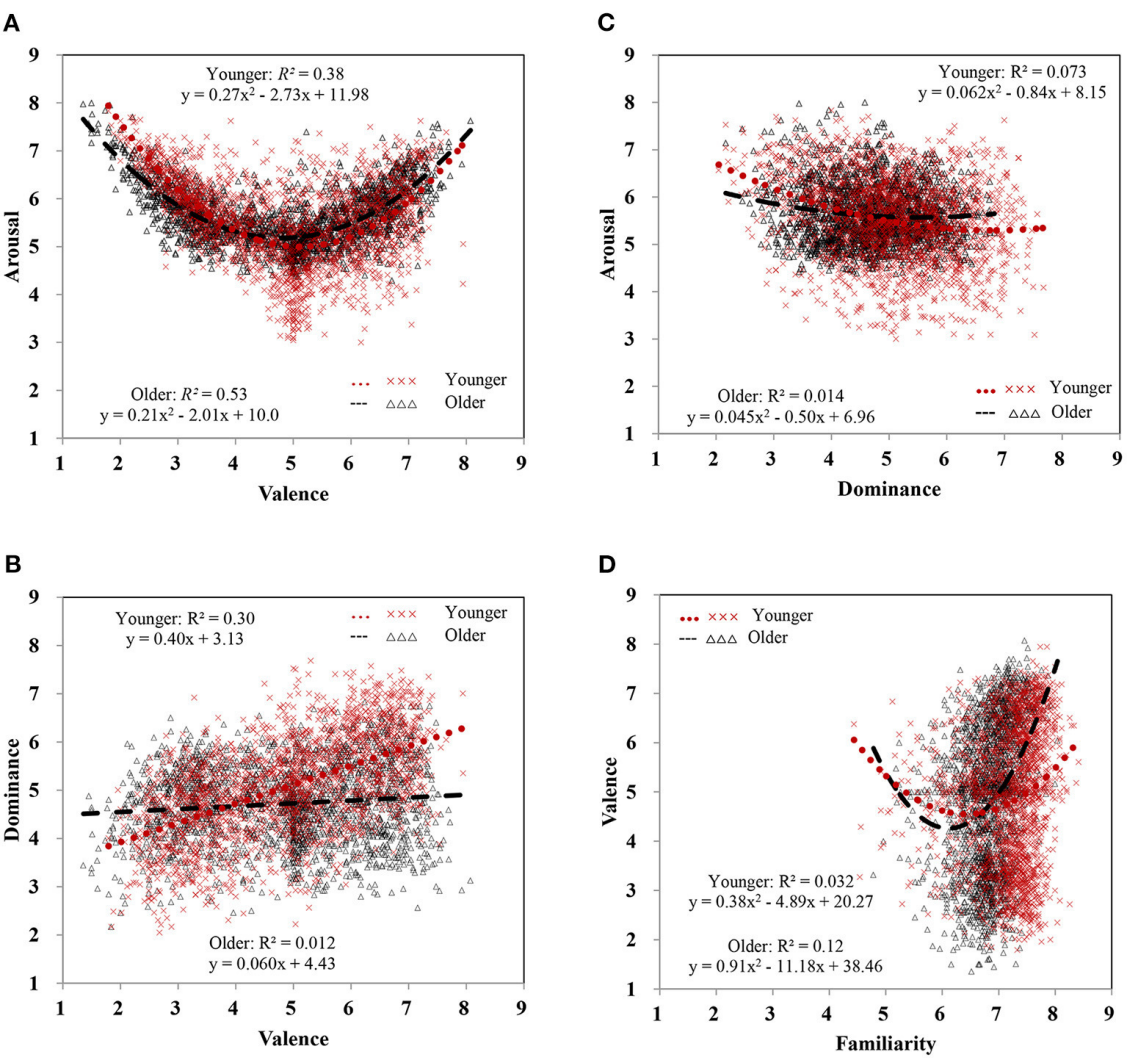
Age differences in the scatterplots of dimensions [**(A)**, valence vs. arousal; **(B)**, valence vs. dominance; **(C)**, dominance vs. arousal; **(D)**, familiarity vs. valence] for all 2,061 words. The best fit of regression lines between dimensions is shown by black dashed and red dotted lines for older and younger adults, respectively.

Figure 6 shows the location of each word in a two-dimensional space defined by the mean ratings of each word. These age-related differences yielded the following patterns. First, compared to younger adults, older adults tended to rate negative words (*M*_*valence*_ < 4.27) as less arousing and more in control (Figures 6A,B) and positive words (4.27 < *M*_*valence*_ < 7.73) as more exciting and less dominant. Second, compared to older adults (*r*_*older*_ = 0.11), younger adults had a stronger tendency (*r*_*younger*_ = 0.55, *p* < 0.001) to rate positive words as more in control than negative words (Figure 6B). Third, there were negative correlations between dominance and arousal (*r*_*older*_ = −0.10, *r*_*younger*_ = −0.25, *ps* < 0.001). Older adults tended to rate those higher dominant words (*M*_*dominance*_ > 3.82) as more arousing, while younger adults tended to rate lower dominant words as more arousing (Figure 6C). Fourth, older adults showed a stronger positive relationship between familiarity and valence than younger adults did (*r*_*older*_ = 0.30, *r*_*younger*_ = 0.12, *ps* < 0.001), and they tended to rate more familiar words (*M*_*familiarity*_ > 6.88) as more positive (Figure 6D). While pinning down the nature of these age-related differences will be an issue for further investigation, these valuable age-related differences in emotional rating should be considered as potential sources of systematic error or bias for research into affective words.

## Age-Related Differences in Ratings

In order to obtain more insights from our data regarding the impact of age, we performed several analyses. First, we grouped the 2,061 words into negative (*M*_valence_ ≤ 4), neutral (4 < *M*_valence_ ≤ 6), and positive words (*M*_valence_ > 6) on the basis of the overall valence score, according to the same criteria used in prior studies (Warriner et al., 2013; Yao et al., 2017). This procedure resulted in 644 negative, 867 neutral, and 550 positive words. Please note that this grouping was on the basis of the overall valence score. Some words might change their polarity (negative, neutral, and positive) when they were considered under different age groups. For example, the word 期中考试(/qi2zhong1kao3shi4/, midterm exams) was rated as neutral based on the overall (*M*_*valence*_ = 4.20) and older adults' rating valence score (*M*_*valence*_ = 5.41) with no change to the neural label. However, younger adults considered it as a negative word (*M*_*valence*_ = 2.72). Therefore, we checked the list and removed 276 words that belonged to different valence categories (polarity) in the two age groups. This procedure resulted in 1,785 words, including 583 negative, 716 neutral, and 486 positive words.

**Figure 7:**
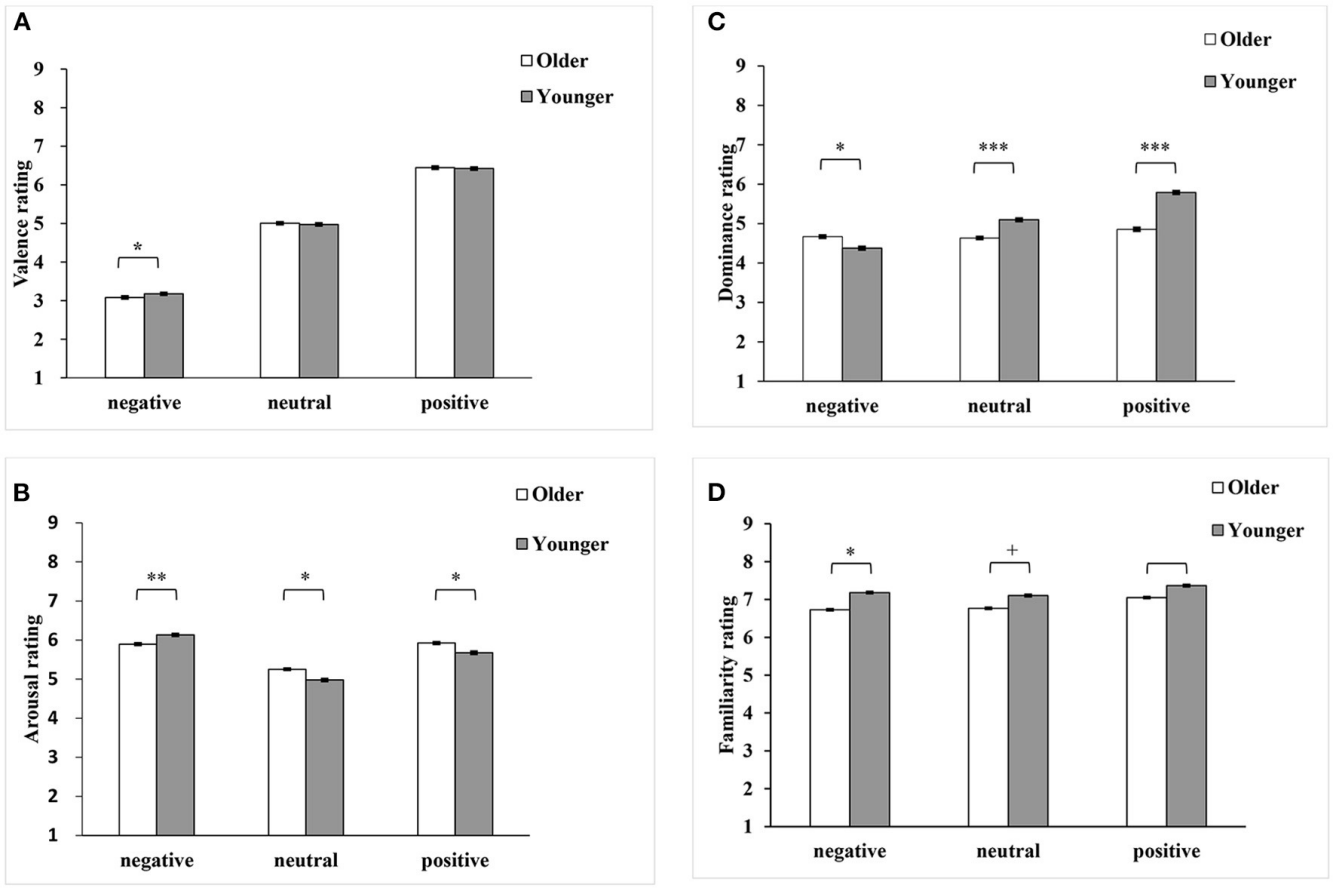
Means and standard errors of ratings in the four emotional dimensions [**(A)**, valence; **(B)**, arousal; **(C)**, dominance; **(D)**, familiarity] for older and younger adults as a function of polarity (negative, neutral, and positive). ***p < 0.001, **p < 0.01, *p < 0.05, ^+^0.05 < *p* < 0.10.

Second, we analyzed ratings (valence, arousal, dominance, and familiarity) as a function of the age group (older vs. younger) and polarity (negative, neutral, and positive) using linear mixed-effects model (LMM). The age group and polarity were entered as fixed effects, specifying the participants and items as cross random effects (Baayen et al., 2008). The *lmer* function was obtained from the *lme4* package (Bates et al., 2014) in *R* statistical software (R Core Team, 2019). As shown in Table 4 and Figure 7, the interactions between age group and polarity, and the main effects of polarity (except dominance dimension), and the main effects of age were significant for the four dimensions. Further analyses indicated that older adults tended to rate positive words as more arousing (*b* = 0.29, *SE* = 0.13, *t* = 2.19, *p* = 0.030) and less controllable (*b* = −0.83, *SE* = 0.14, *t* = −5.98, *p* < 0.001) than younger adults did. Older adults also tended to rate neutral words more arousing (*b* = 0.22, *SE* = 0.088, *t* = 2.47, *p* = 0.014) and less controllable (*b* = −0.37, *SE* = 0.10, *t* = −3.51, *p* < 0.001). In contrast, older adults tended to rate negative words as more negative (*b* = −0.081, *SE* = 0.037, *t* = −2.23, *p* = 0.027), less arousing (*b* = −0.34, *SE* = 0.11, *t* = −3.03, *p* = 0.0027), more controllable (*b* = 0.35, *SE* = 0.16, *t* = 2.12, *p* = 0.037), and less familiar (*b* = −0.41, *SE* = 0.17, *t* = −2.45, *p* = 0.016) than younger adults did. These results revealed that older adults attended to positive information to a greater extent than younger adults, and this supported the age-related positivity effect. We also performed the *LMM* analysis for the whole 2,061 words and obtained similar statistical conclusions.

**Table 4:** Results of the linear mixed models analysis for ratings (valence, arousal, dominance, and familiarity) as a function of age group (older vs. younger) and polarity (negative, neutral, and positive).

**Fixed effects**	***b***	***SE***	***t***	***p***	**95% CI**
**Valence**					
Intercept	2.99	0.026	116.78	<0.001	[2.934, 3.035]
Age	0.081	0.037	2.23	0.027	[0.009, 0.153]
Polarity	2.08	0.012	176.42	<0.001	[2.060, 2.106]
Age × Polarity	−0.13	0.017	−7.98	<0.001	[−0.166, −0.100]
**Arousal**					
Intercept	5.84	0.079	74.07	<0.001	[5.686, 5.996]
Age	0.34	0.11	3.03	0.003	[0.119, 0.561]
Polarity	−0.65	0.076	−8.57	<0.001	[−0.804, −0.504]
Age × Polarity	−0.56	0.11	−5.10	<0.001	[−0.771, −0.341]
**Dominance**					
Intercept	4.70	0.13	37.54	<0.001	[4.451, 4.947]
Age	−0.35	0.16	−2.12	0.037	[−0.673, −0.022]
Polarity	0.19	0.13	1.49	0.140	[−0.062, 0.435]
Age × Polarity	1.18	0.17	7.14	<0.001	[0.850, 1.503]
**Familiarity**					
Intercept	6.71	0.13	52.76	<0.001	[6.453, 6.956]
Age	0.401	0.17	2.45	0.016	[0.078, 0.738]
Polarity	0.051	0.013	4.06	<0.001	[0.026, 0.075]
Age × Polarity	−0.13	0.018	−7.13	<0.001	[−0.161, −0.092]

*CI, confidence interval*.

Finally, according to the analyses introduced by Grühn and Smith (2008), we explored age-related differences in the perception of individual words. We conducted univariate ANOVAs for individual word with age as a between-subjects factor. This procedure resulted in 4 (dimensions) × 2,061 (words) = 8,244 analyses on the univariate level. Here, 756 words (36%) showed no differences for all four dimensions. However, there were a substantial number of significant main effects of age for valence (413 words), arousal (418), dominance (566), and familiarity (525). These robust age-related differences were shown mainly from neutral words for valence (197; 48% of 413 words with significant age effects), arousal (212; 51% of 418 words), and familiarity dimensions (230; 44% of 525 words). We found the main discrepancies between the ratings of older and younger adults came from the value, attitudes, and life experience. The nature of these age-related differences in ratings needs to be investigated in future studies.

## General Discussion

The goal of this study was to establish the AANC database and make these age-related ratings available in the public domain. Although there is a growing body of aging-oriented research on emotion and language, no published research on affective norms for older adults are available in China. Meanwhile, many studies frequently use ratings of younger adults to classify stimuli for both older and younger adults. This would not have taken into account the potential age-related differences in the perception of material. To address this issue, our work provides valence, arousal, dominance, and familiarity ratings of older and younger adults for 2,061 four-character words in Chinese. With regard to participants' age, the AANC database shows consensus and variation in the perception and meaning of affective words. We found further evidence of the age-related positivity effect, as older adults indeed had preference for positive over negative stimuli relative to younger adults. To our knowledge, our AANC database has been the largest published database reporting older adults' assessments of the emotional properties of words so far. This resource will enable researchers to study how emotional words influence cognitive processing and how this influence evolves with age. Furthermore, the large-scale database has great value as a resource for automated affective analysis in natural language processing applications (Kratzwald et al., 2018; Liu et al., 2018).

## Correlations Between Dimensions

Consistent with previous research, this study also shows strong correlations between different dimensions. First, we found the typical U-shaped relationship between valence and arousal. Very positive and very negative words were typically evaluated as highly arousing, whereas less emotional and neutral words were less arousing (Bradley and Lang, 1999; Wang et al., 2008; Eilola and Havelka, 2010; Soares et al., 2012; Warriner et al., 2013; Schmidtke et al., 2014; Stadthagen-Gonzalez et al., 2017; Yao et al., 2017; Liu et al., 2018). Second, our results demonstrated that dominance was positively related to valence (Grühn and Smith, 2008; Warriner et al., 2013; Fairfield et al., 2017), indicating that positive words were more controllable than negative words. Third, we found that dominance was negatively related to arousal, indicating that words considered less dominant were more arousing (Schmidtke et al., 2014). Fourth, familiarity was positively associated with valence, arousal, and dominance. Words rated as more familiar were likely to be regarded as more positive, exciting, and strong. Familiarity ratings have been interpreted as a measure on the frequency of exposure to a word (Stadthagen-Gonzalez and Davis, 2006; Eilola and Havelka, 2010). These might be related to the exposure effect (Zajonc, 1968) or familiarity effect (Warriner et al., 2013; Sabater et al., 2020). Repeated exposure of people to these stimuli (familiarity) enhances their positive attitude toward them, such as preferences, positivity bias, and control.

The strength of the correlations between different dimensions may have some implications from the dimensional perspective of emotion, since the original model assumes that three dimensions of emotion are orthogonal (Wundt, 1912/1924; Osgood et al., 1957; Russell, 2003). More specifically, the operationalization of dominance may be more complex than previously thought. Even though dominance has been identified as an important variable in emotion research, it was not often included in previous word norming studies (Gilet et al., 2012; Monnier and Syssau, 2017; Stadthagen-Gonzalez et al., 2017; Yao et al., 2017; Liu et al., 2018). We found that dominance and valence were strongly related, and this may point to the utility of considering valence/dominance strength (i.e., how different a word is from neutral) or polarity as the explanatory variable (Warriner et al., 2013). It is unclear at this time whether the three affective dimensions could probably be reduced to two latent dimensions (Fontaine et al., 2007; Grühn and Smith, 2008). Future studies need to validate that dominance explains unique variance in emotional information processing. Our database provides the raw data for future studies in the dimensional perspective of emotion modeling (e.g., Russell, 2003; Fontaine et al., 2007).

## The Impact of Age: Consensus and Variations

With regard to the impact of age, this study shows two major findings. First, we found that the age-related positivity effect was related to older adults' preference for pleasant stimuli. Older adults tended to evaluate positive words as more arousing and less controllable than younger adults did. In contrast, they tended to rate negative words as less arousing and more controllable than younger adults did. These findings are consistent with results reported by Grühn and Smith (2008). We also found a stronger relationship between valence and arousal for younger adults than that for older adults, which is inconsistent with some prior studies (Gilet et al., 2012; Ready et al., 2017). These inconsistencies may indicate that the emotional meanings of some words vary with languages. These discrepancies could also be due to other factors. Older adults may be especially motivated by goals related to emotional satisfaction according to the context of socioemotional selectivity theory (Carstensen, 2006; English and Carstensen, 2014; Reed et al., 2014), and they showed an information processing shift toward positive information in later life. These age-related differences may also be a function of life experience, lifetime exposure, cultural environments, or age-related changes in psychological, biological, and social functioning.

Second, the two age groups agreed on the pleasantness of words, as was evident from their high correlation on valence (although not for the other three dimensions). The two groups agreed on whether a word was positive or negative. Unlike the findings reported by Grühn and Smith (2008), we did not find that older adults tended to evaluate positive words more positively and negative words more negatively than that of younger adults. These discrepancies may result from different stimuli, cultures, or statistical analyses, which need to be investigated in future research. Additionally, we found that the perceived valence of words tended to generalize well, in line with prior studies (Eilola and Havelka, 2010; Soares et al., 2012; Moors et al., 2013; Warriner et al., 2013). However, the ratings of arousal, dominance, and familiarity showed greater variability across older and younger adults. Younger adults rated words significantly higher for dominance and familiarity than older adults.

### Summary, Limitations, and Conclusion

This study provides a large-scale database for four-character words in Chinese, which clearly demonstrates age-related differences in affective norms. Although some rating results were consistent between younger and older adults, there were still some differences in ratings for a large number of words. In general, older adults tended to rate positive words as more arousing and less controllable and negative words as less arousing and more controllable than younger adults did. Overall, older adults tended to give more extreme valence ratings to the words than younger adults did, whereas younger adults tended to rate emotional words as more controllable and familiar than older adults did. These results indicate that the positivity effect is reliable and older adults prefer positive stimuli.

We provide an age-adopted tool for future research on the processing of emotional words from a developmental point of view. However, there are some limitations in the present study. First, our materials did not contain two- or three-character words due to our limitations of time and funds. Second, this paper purposely did not include detailed analysis on gender differences because there were small samples of each gender for each age group. Finally, many studies suggest that discrete emotions (e.g., happiness, anger, fear, disgust, guilt, and sadness) play a role in information processing that goes beyond valence and arousal (Ferré et al., 2017; Stadthagen-González et al., 2018). Future studies could expand the database to include gender differences and discrete emotional categories for large sets of words including both two-, three-, and four-character words.

In sum, our data set provides a useful resource for studies in which the effects of aging are considered and affective words are used. The collection of affective norms for 2,061 Chinese words will give computational and experimental researchers a much wider selection of materials for their studies. Using the AANC word pool, researchers can study how affective states of the words influence the cognitive processes and how this influence evolves with age.

## Data Availability Statement

The original contributions presented in the study are included in the article/Supplementary Material, further inquiries can be directed to the corresponding author/s.

## Ethics Statement

The studies involving human participants were reviewed and approved by the Institutional Review Board of the Institute of Psychology, Chinese Academy of Sciences. The patients/participants provided their written informed consent to participate in this study.

## Author Contributions

PL designed the study. PL and ZZ assembled data. PL and JT analyzed the data, PL wrote the paper. PL, QL, ZZ, JT, and BH revised the paper. All authors contributed to the article and approved the submitted version.

## Conflict of Interest

The authors declare that the research was conducted in the absence of any commercial or financial relationships that could be construed as a potential conflict of interest.
