# Corrigendum: Age-related differences in affective norms for Chinese words (AANC)

**DOI:** 10.3389/fpsyg.2023.1303942

**Published:** 2023-11-08

**Authors:** Pingping Liu, Qin Lu, Zhen Zhang, Jie Tang, Buxin Han

**Affiliations:** ^1^CAS(Chinese Academy of Sciences) Key Laboratory of Mental Health, Center on Aging Psychology, Institute of Psychology, Beijing, China; ^2^Department of Psychology, University of Chinese Academy of Sciences, Beijing, China; ^3^Department of Computing, The Hong Kong Polytechnic University, Hong Kong, China

**Keywords:** affective norms, ratings, age differences, Chinese, positivity effect

In the published article, there was an error in Supplementary material “Presentation 1.” The Chinese version “其中, 2=非常不平静, 3=比较不平静, 4=有点不平静” is wrong. It should be “其中, 2=非常平静, 3=比较平静, 4=有点平静.” That is, the Chinese character “不” should be deleted.

The authors apologize for this error and state that this does not change the scientific conclusions of the article in any way. The original article has been updated.

