# Supplementary material for: Age-Related Differences in Affective Norms for Chinese Words (AANC)
Source: Front Psychol. 2021 Apr 15;12:585666. doi: 10.3389/fpsyg.2021.585666 (PMC8082186; doi:10.3389/fpsyg.2021.585666)
Supplement: Supplementary file 2 [file Presentation_1.pdf]

Appendix: Instructions

The original Chinese instructions are presented as well as their English translation.

欢迎您参与本研究！请根据您的第一感觉和经验来评价词语带给您的感受。请您在9点量表上对同一个词进行两步评价，用鼠标左键准确点击最能代表您直观感受的相应数字。选择没有正确错误之分，没有时间限制，但是请您不要花费太多时间考虑这些词。

You are invited to take part in the study that is investigating how people respond to different types of words. Your will use a 9-point scale to rate how you felt while reading each word in two steps. There were no right and wrong answers, and the best answer would reflect your true opinion about the word. Please make your ratings based on your first and immediate reactions by clicking on the appropriate figure using a computer mouse. Please work at your own pace and don't spend too much time thinking about each word.

Instructions for rating valence and arousal (Adapted from Bradley & Lang, 1999; Stadthagen-Gonzalez et al., 2017; Wang et al., 2008; Warriner et al. 2013)

第一步：请您评价看到词语后的愉快程度，点击一下相应数字。1表示极其不悦、极其烦恼、极度不满意、很忧伤或非常失望等负性感受。9表示极其高兴、极其愉快、极其满意或充满希望等正性感受或正能量。其中，2=非常不愉悦，3=比较不愉悦，4=有点不愉悦，5=感觉一般，没有任何负性或者正性的感受，6=有点愉悦，7=比较愉悦，8=非常愉悦。

第二步：请评价您看到同一个词后的心情激动程度，点击一下相应数字。1表示看词后感到极其平静放松、不警觉、极少刺激性、引起的关注量最少等。9表示看

词后感到极其激动、够刺激、令人非常觉醒、极其兴奋、眼前一亮或者心情起伏波动非常大等。其中，2=非常平静，3=比较平静，4=有点平静，5=感觉一般，6=有点激动，7=比较激动，8=非常激动。

In the first step, please judge the extent to which the words referred to something that is positive/pleasant or negative/unpleasant using a 9-point scale. At one extreme of the scale, you are completely unhappy, annoyed, unsatisfied, melancholic, despaired or bored. When a word makes you feel extremely unhappy, you should indicate it by selecting 1. The other end of the scale is for when you feel extremely happy, pleased, satisfied, contented, or hopeful. When a word makes you feel extremely happy, you should indicate it by selecting 9. The other numbers on the scale also allow you to describe your intermediate feelings of pleasure when you read each word (2=very negative/unpleasant, 3=moderately negative/unpleasant, 4=slightly negative/unpleasant, 5=neither happy nor sad, 6=slightly positive/pleasant, 7=moderately positive/pleasant, 8= very positive/pleasant).

In the second step, please judge the extent to which the words referred to something that is calm or excited using a 9-point scale. At one extreme of the scale, you are completely relaxed, calm, sluggish, dull, or sleepy. When a word makes you feel totally calm, you should indicate it by selecting 1. The other end of the scale is for when you feel stimulated, excited, frenzied, jittery, wide-awake, or aroused. When a word makes you feel totally excited, you should indicate it by selecting 9. The other numbers on the scale also allow you to describe intermediate feelings of calmness/arousal (2=very calm, 3=moderately calm, 4=slightly calm, 5=neither calm nor excited, 6=slightly excited, 7=moderately excited, 8=very excited).

Instructions for rating dominance and familiarity (Adapted from Bradley & Lang, 1999; Wang et al., 2008; Warriner et al. 2013)

第一步：请您评价看到词语后的控制支配感。从1到9表示控制感越来越强。1表示感到自己完全地受控制、受影响、感到敬畏、感到弱小顺从、被引导、受约束、受操控的、屈服的、无能为力的。9表示感到自己完全处于支配地位、具有充分的支配权和控制感，完全能制约的、能操纵的、有影响力的、有重要地位的、居优势地位的、有自主权的。5表示一般，即您感到既不受控制，也没有感受到支配地位，您可以选择“5”。其中，2=非常受支配，3=比较受支配，4=有点受支配，5=感觉一般，6=有点控制感，7=比较控制感，8=非常控制感。

第二步；请您评价对于这个词汇的熟悉程度，从1到9熟悉度越来越强。1表示阅读词汇后感到强烈的陌生和生疏感、不认识这个词，从来没有见过、看过或者用过这个词。9表示对这个词极其的熟悉、熟知、亲切、经常使用等，比如通过语言交流、网络、电视和书籍等多种方式见过。其中，2=非常不熟悉，3=比较不熟悉，4=有点不熟悉，5=感觉一般，6=有点熟悉，7=比较熟悉，8=非常熟悉。比如看到词汇“自贻伊戚”，您可能从来没有见过，就可以选择数字“1”；而看到词汇“美味米饭”，您可能每天都见到。

In the first step, please judge the extent to which the words referred to something that is weak/submissive or strong/dominant using a 9-point scale. At one extreme of the scale, you are completely influenced, cared-for, awed, submissive, or guided. When a word makes you feel completely submissive, you should indicate it by selecting 1. The other end of the scale is for when you feel extremely in control, influential, important, dominant, autonomous, or controlling. When a word makes you feel completely dominant, you should indicate it by selecting 9. The other numbers on the scale also allow you to

describe your intermediate feelings of control when you read each word (2=very weak/submissive, 3=moderately weak/submissive, 4=slightly weak/submissive, 5=neither submissive nor dominant, 6=slightly strong/dominant, 7= moderately strong/dominant, 8= very strong/dominant).

In the second step, please judge the familiarity which involves rating how often the given word occurs in everyday language in either written or spoken form using a 9-point scale. At one extreme of the scale, you are completely familiar with this word in everyday language. Maybe you often hear the word on conversation, at the radio, at TV, or you may find it in a written form books, Internet, etc. When a word makes you feel totally familiar, you should indicate it by selecting 9. Conversely, a sore of 1 indicates that you rarely find the word in everyday language. The other numbers on the scale also allow you to describe intermediate feelings of familiarity (2=very unfamiliar, 3=moderately unfamiliar, 4=slightly unfamiliar, 5=undecided, 6=slightly familiar, 7= moderately familiar, 8= very familiar). For example, the word 美味米饭 (/mei3wei4mi3fan4/, delicious rice) could be rated as occurring in everyday language very often, whereas the word 自贻伊戚 (/zi4yi2yi1qi1/, torture oneself) could be rated as never occurring in everyday language.

最后再举例：比如看到词汇“国家军队”、“打架斗殴”、“蓝天白云”、“桌子椅子”等。请问按照您的直觉体验感受，在相应维度上会选择哪个数字？

Finally, we provide examples (e.g., national army, fight and hit, blue sky and white clouds, tables and chairs) for the ratings of relevant dimensions. According to your first and immediate reactions, which figure would you select?
